# Illness Perception Predicting Cardiovascular Health Behaviors among Patients with Ischemic Heart Disease in Nepal: A Descriptive Cross-sectional Study

**DOI:** 10.31729/jnma.5426

**Published:** 2020-11-30

**Authors:** Punam Gauro, Ploenpit Thaniwattananon, Charuwan Kritpracha

**Affiliations:** 1 Faculty of Nursing, Prince of Songkhla University, Hatyai, Thailand

**Keywords:** *cardiovascular health behaviors*, *illness perception*, *ischemic heart disease*

## Abstract

**Introduction:**

Cardiovascular health behaviors refer to the activities done by individuals to prevent recurrence, minimize risk factors, improve survival, reduce recurrent events, control cardiovascular disease, and help prevent further complications. Illness perception may determine these behaviors. This study is aimed to identify level of illness perception, cardiovascular health behaviors and illness perception predicting cardiovascular health behaviors among patients with ischemic heart disease.

**Methods:**

The study was a descriptive cross-sectional study. Altogether 114 samples were selected using convenience sampling technique. Data was collected by using pretested semi-structured questionnaire. The data were analyzed using descriptive statistics.

**Results:**

Illness perception was at moderate level (M= 148.05, SD= 12.86) which represented a moderate threatening perception. The score of cardiovascular health behaviors was at a high level (M=92.14, SD=10.72).

**Conclusions:**

The findings showed that illness perception can be a predictor of cardiovascular health behaviors.

## INTRODUCTION

Worldwide cardiovascular disease (CVD) is the leading cause of mortality. It has been reported that 17.9 million people died from CVD in 2016, constituting 31% of all global deaths^1^ and half of these is estimated to occur in Asia.^2^ Among CVD, ischemic heart disease (IHD) is one of the main causes of mortality.^1^

Illness perception was also identified as a factor related to individual cardiovascular health behavior.^3^ Most patients with IHD have less knowledge about the importance of cardiovascular health behaviors to prevent recurrence of IHD.^4^ Thus, patients with diagnosed IHD remain at high risk for cardiac event reoccurrence.^5^ Each individual could intensely change their cardiovascular health behaviors according to their perception^6^ using systematic sampling, the first 6 patients with type 2 diabetes mellitus aged > or =18 years who visited them for consultation during the study period. Data on 250 patients included patient illness perception assessment (Brief Illness Perception Questionnaire, IPQ).

The existing knowledge regarding illness perception and cardiovascular health behaviors among IHD patients is limited in Nepal.^7^ This study aimed to examine the level of illness perception, cardiovascular health behaviors, and illness perception predicting cardiovascular health behaviors among IHD patients.

## METHODS

This is a descriptive cross-sectional study. This study was conducted in a public hospital (BP Koirala Institute of Health Sciences, Dharan) in Eastern Nepal from January to March 2018. Prior to the commencement of the study the researcher obtained approval from the Institutional Review Board (IRB), University of Thailand and Nepal Health Research Council (Reference No 1672) and BPKIHS, Dharan. All the participants also gave their informed written consent. Common Sense Model (CSM) of illness perception proposed by Leventhal et al.^8^ is used as conceptual framework. Patients aged more than 18 years, able to communicate, understand Nepali language and diagnosed with stable IHD were included.

A convenience sampling method was used. The total participants were 114 outpatients. The sample size was calculated as below:

$$\begin{array}{ll} \text{n} & \text{= }{\text{Z}}^{\text{2}}\times \text{p}\times (\text{1}-\text{p)}/{\text{e}}^{\text{2}}\\ & \text{= }{\left(1.64\right)}^{\text{2}}\times 0.5\times (1-0.5)/{(\text{0.1)}}^{\text{2}}\\ & \text{= }67 \end{array}$$

Where,

n = required sample size
Z = Confidence Interval (CI), 90%
p = prevalence, 50%
e = Margin of error, 10%

Therefore, the calculated sample size was 67. However the sample taken was 114 people diagnosed with IHD. The researcher collected data by interviewing with a standardized instrument. There searcher employed three instruments: (1) Demographic Data and Health Related Information (DHI), (2) Illness Perception Questionnaire-Revised (IPQ-R), and (3) The Modified Cardiac Health Behaviors Scale (MCHBS). All the questionnaires were in English which were later translated into the Nepali language. Three experts validated the content validity of the instruments. Two experts from Prince of Songkla University and one from Nepal. The test-retest reliability was tested for IPQ-R and MCHBS.

Demographic Data and Health Related Information (DHI) was a self-constructed questionnaire. DHI includes age, gender, marital status, education, and occupation. Illness perception was measured using Illness Perception Questionnaire-Revised developed by Moss-Morris.^10^ The cardiovascular health behavior scale was developed by Oh et al.^11^ Although the questionnaire is relevant to this study, some of the items are not specific. Therefore, the researcher modified the questionnaire to assess cardiac health behaviors based on the American Heart Association (AHA) guideline.

Data collected was be kept in Microsoft Excel and then edited and checked. After that the data will be put in SPSS, the descriptive statistical analysis was done to describe DHI. Frequency, percentages will be calculated for binary data and mean and standard deviation will be calculated for continuous data after the normality of the data has been checked.

## RESULTS

The results from this study showed that participants were between the ages of 34 and 74 (M= 52.84, SD= 8.24) and majority of the participants 45 (39.5%) were in the 51 to 60 age group. More than half of the patients 67 (58.8%) were male and most of them were Hindu 82 (55%). Many patients 45 (39%) completed a secondary level of education and only 9 (7.9%) of the patients were unemployed. Majority of the patients 98 (86%) were from extended family and two-thirds of the patients 80 (70.1%) had a monthly income of 10,000 to 30,000 Nepalese rupees (NPR) (approx. 1 US dollars = 108 Nepali rupees).

The mean body mass index (BMI) was 26.04 kg/m2 (SD= 2.97) and the duration of illness in most of the patients 74 (64.9%) with IHD in this study was more than 12 months. Majority of the patients 79 (69.2%) had a history of smoking. Majority of the patients 100 (87.7%) reported that hypertension was their comorbidity. Medications recently used by the patients were antihypertensive drugs, anticoagulants and aspirin, and antidiuretics in 10 (87.7%), 88 (77.2%), and 29 (25.4%) of the patients respectively (Table 1).

The total illness perception mean score among the patients with IHD was M = 148.05 (SD = 12.86)

**Table 1:** Health related characteristics of patients with IHD (n = 114).

Characteristics	n (%)
BMI, kg/m^2^ (M = 26.04, SD = 2.97, Min = 18.03, Max = 33.69)	
<18.5 (Underweight)	1 (0.9)
18.5-24.9 (Normal)	43 (38.7)
25-29.9 (Overweight)	60 (51.4)
≥30 (Obese)	10 (9)
Duration (M = 3.32, SD = 0.973, Min<1, Max≥12 months)	
<1	
1-6	4 (3.5)
7-12	29 (25.4)
>12	7 (6.1)
History of Smoking	
Smoker	74 (64.9)
Non-smoker	79 (69.2)
Comorbidity^a^	
Diabetes Mellitus	35 (30.8)
Hypertension	83 (72.8)
Others	10 (87.7)
CA cervix	1 (0.9)
Gastritis	18 (16.2)
Medications used^a^	
Antihypertensive	10 (87.7)
Anticoagulant and aspirin	88 (77.2)
Antidiabetic	29 (25.4)

Note M = Mean; SD = Standard deviation; Min = Minimum; Max = Maximum

Note: ^a^Patients provided more than one answer.

which indicated moderate threatening perception of the illness. The dimension consequences had the highest level (M= 22.29, SD= 3.09). Other dimensions reported moderate levels which were timeline acute/ chronic (M= 21.75, SD= 3.76), timeline cyclical (M= 13.64, SD= 2.01), concern (M= 13.26, SD= 2.93), personal control (M= 20.32, SD= 2.40), treatment control (M= 17.40, SD= 2.08), emotion (M= 21.92, SD= 3.24), and coherence (M= 17.40, SD= 2.08) (Table 2).

**Table 2:** Level of illness perception among patients with IHD (n=114).

Variable	Dimensions	Min	Max	M	SD	Level
Illness Perception	Total Score	121	183	148.05	12.86	Moderate
	Timeline acute/chronic	11	30	21.75	3.76	Moderate
	Timeline cyclical	4	19	13.64	2.01	Moderate
	Consequences	15	30	22.29	3.09	High
	Coherence	11	22	17.40	2.08	Moderate
	Personal Control	14	26	20.32	2.40	Moderate
	Treatment Control	11	22	17.40	2.08	Moderate
	Concern	8	20	13.26	2.93	Moderate
	Emotion	12	29	21.92	3.24	Moderate

Note M = Mean; SD = Standard deviation; Min = Minimum; Max = Maximum

The perceived causes were smoking 62 (54.3%), followed by diet 58 (50.8%), stress 57 (50%), ageing 48 (42.10%), heredity 34 (28.07%), alcohol 34 (28.07%), hypertension 26 (22.80%), physical inactivity 26 (22.80%), and diabetes mellitus 10 (8.77%) (Table 3).

**Table 3:** Perceived causes by IHD patients (n=114).

Causes	n (%)
Smoking	62 (54.3)
Diet	58 (50.8)
Stress	57 (50)
Ageing	48 (42.1)
Heredity	34 (28.1)
Alcohol	34 (28.1)
Hypertension	26 (22.8)
Physical inactivity	26 (22.8)
Diabetes Mellitus	10 (8.8)

Participants reported a high level of cardio vascular health behaviors (M= 92.14, SD= 10.72). The dimensions of cardiovascular health behaviors in IHD patients were reported at high levels of taking medications (M= 11.60, SD= 0.72) and blood pressure control (M= 15.73, SD= 3.71). The dimensions of cardiovascular health behaviors that the patients reported to perform at moderate levels were diet management (M= 25.04, SD= 4.12), physical activity (M= 13.72, SD= 2.65), smoking cessation (M= 11.61, SD= 3.52), and stress management (M= 11.04, SD= 2.24) (Table 4).

**Table 4:** Level of cardiovascular health behaviors among patients with IHD (n=114).

Variable Dimensions	Min	Max	M	SD	Level
Cardiovascular	Total Score	67	113	92.14	10.72	High
Health Behaviors	Smoking Cessation	8	16	11.61	3.52	Moderate
	Diet management	15	32	25.04	4.12	Moderate
	Blood pressure control	8	20	15.73	3.71	High
	Physical activity	5	20	13.72	2.65	Moderate
	Stress management	6	15	11.04	2.24	Moderate
	Taking medications	8	12	11.60	0.72	High

Note M = Mean; SD = Standard deviation; Min = Minimum; Max = Maximum

## DISCUSSION

The mean age of the patients in this study ranged between 50-60 which is similar to previous studies.^5^ The majority of the IHD patients were male, which was similar to the previous studies on IHD that reported more male patients in Nepal^12^ and in a Jordanian study.^5^ In this study, the causes of IHD tended toward smoking as a lifestyle factor because majority of the participants perceived that smoking causes IHD. A previous study reported that smoking by more males than females was one of the factors reported to cause IHD.^13^ Majority of patients were hypertensive and most of them were overweight. It has been reported in previous study that hypertension was common among obese patients and could trigger IHD and it was also the major co-morbidity occurring in IHD patients.^5^

In this study majority of the patients perceived that the major causes of their disease IHD were smoking, diet and stress. The causes were similar with previous studies.^5^ The level of cardiovascular health behaviors among IHD patients was high in this study. This indicated that the patients with IHD had high performance of cardiovascular health behaviors. The reason behind the high level of cardiovascular health behavior of IHD patients was possibly due to the early health education received by the patients and other counseling from health personnel on smoking cessation, diet, exercise, taking medicines on time, and stress management. As a result, they had better perception of their illness. Therefore, this made the patients aware of their health. One study revealed that training and education helped IHD patients for better health outcome.^14^

The high level of cardiovascular health behaviors was beneficial for the IHD patients, as this showed they were conscious and aware of their health conditions. The dimensions of cardiovascular health behaviors by the IHD patients were at a high level for taking medications and blood pressure control. One of the studies^15^ found that family and support from friends helped them changed their lifestyles and also resulted in changing the cardiovascular health behaviors. In Nepal, support from family members was one of the most important and strongest support systems for their whole lives.

This study found that diet management, physical activities, smoking cessation and stress management of the patient's behavior was at a moderate level. The IHD patients in Nepal perceived that many cultural practices might influence the management of diet in IHD patients to take different varieties of food which contain high calories. There are many festivals which influence the intake of high calorie food.^7^ One previous study had revealed that a positive attitude helped to improve cardiovascular health behaviors and prevented the risk.^16^

The relationship between illness perception and cardiovascular health behaviors among IHD patients in this study revealed a negative correlation with a significant level of 0.05. This finding was in contrast with previous studies^17^ as they found positively significant association between illness perception and cardiovascular health behaviors. Likewise, previous studies on illness perception involving patients with other chronic disease, such as diabetes, asthma, and osteoarthritis, found that illness perception were strong predictors of cardiovascular health behaviors.^18^

There might be several reasons behind the negative relationship. Illness perception of the patients towards IHD changed during rehabilitation following the first attack.^19^ These changes of illness perception were associated with the proper cardiovascular health behaviors which helped to prevent recurrence of IHD and enhanced their better health.^19^ Another study found that participating in cardiac rehabilitation (CR) could increase blood circulation and maintain the oxygen level that resulted in a significant increase in the functional heart capacity and a significant reduction in the heart rate.^20^ Knowledge of the illness and adjustment might have reduced the negative perception of illness and increased cardiovascular health behavior. The majority of the patients in this study were educated. Kang et al reported that persons with a higher education were more capable of understanding the necessity of changing their lifestyle which motivated them to perform cardiovascular health behaviors more often.^4^

Majority of the patients perceived illness identity as chest pain; breath lessens, and body discomfort. For these symptoms, the patients perceived to take good suggestions from the health care providers. The findings were similar with previous studies majority of the patients believed that IHD were due to smoking, diet and heredity. The findings were similar with previous study.^5^

The multiple regression analysis revealed that timeline acute/chronic, personal control, and concern were the statistically significant domains strongly predicting cardiovascular health behaviors. Similarly, another study found that personal control was a strong predictor of cardiovascular health behaviors.^21^ Timeline is one of the predictors of cardiovascular health behaviors in this study. The greater time period of diagnosis indicated these patients have received more information regarding their disease condition and cope with illness as well as perform higher cardiovascular health behaviors to adjust with disease. Similarly, personal control predicted cardiovascular health behaviors. The reason personal control could predict cardiovascular health behaviors might be because the majority of the IHD patients in this study were educated, and most of them were already rehabilitated. Limitation of the study was the use of convenient sampling technique during data collection that might reduce generalizability to the population.

## CONCLUSIONS

The study indicated that perception of illness by the patient specially timeline acute or chronic, personal control and concern can predict cardiovascular health behavior. Thus, illness perception of the patient should be taken into consideration while providing education regarding modification of cardiovascular health behavior. This study will be beneficial for the development of knowledge by healthcare professionals to educate IHD patients especially on the misperception and role of illness perception on cardiovascular health behavior.
